# Neural adaptations in short-term learning of sign language revealed by fMRI and DTI

**DOI:** 10.1038/s41598-024-84468-z

**Published:** 2025-02-13

**Authors:** Sahal Alotaibi, Sultan Alamri, Alanood Alsaleh, Georg Meyer

**Affiliations:** 1 Radiological Sciences Department, Applied Medical Sciences, Taif University, Taif, 21944 Kingdom of Saudi Arabia; 2 Faculty of Health and Life Sciences, University of Liverpool, Liverpool, L69 7ZA UK; 3 Radiological Sciences Department, Applied Medical Sciences, King Saud University, Riyadh, Kingdom of Saudi Arabia; 4 Clinical and Cognitive Neuroscience Group, Department of Psychology, University of Liverpool, Liverpool, L69 7ZA UK; 5 Virtual Engineering Centre, Digital Innovation Facility, University of Liverpool, Liverpool, L69 3RF UK; 6 Hanse Wissenschaftskolleg, Lehmkuhlenbusch 4, 27753 Delmenhorst, Germany

**Keywords:** Sign language, Neuroplasticity, IFG, STG, Angular gyrus, Neuroscience, Human behaviour

## Abstract

While vocal articulation is a unique feature of spoken languages, signed languages use facial expressions and hand movements for communication. Despite this substantial difference, neuroimaging studies show that spoken and sign language rely on similar areas of the brain in the frontal and parietal regions. However, little is known about the specific roles of these areas and how early they get involved. In the present study, we investigate the impact of short-term training-related changes in learners of British sign language (BSL). Pre- and post-training functional magnetic resonance imaging (fMRI) and diffusion tensor imaging scans were taken from twenty-six right-handed healthy volunteers. During the training course, participants were taught to discriminate and sign basic sentences using BSL for three consecutive days (1 h per day). fMRI results show increasing brain activity in the right cerebellum and cerebral brain areas including bilateral middle temporal gyrus, left angular gyrus, left middle and inferior frontal gyrus. Moreover, functional connectivity increased significantly after training between these areas. Microstructural findings show significant mean diffusivity and radial diffusivity reductions in the left angular gyrus, which are significantly correlated with behavioural improvement. These results reveal a high degree of similarity in the neural activity underlying signed and spoken languages. The rapid microstructural changes, identify the left angular gyrus as a structure that rapidly adapts to newly learnt visual-semantic associations.

## Introduction

When we think of language, we typically consider spoken and heard communication. An alternative mode of communication is sign language, which, although it uses different articulatory and perceptual systems, draws on the same linguistic structures and largely shared brain areas for language processing (review:^1–3^). Studying the changes that occur when sign language is learnt as a second language affords a unique window into the underlying neural structures and neurocognitive changes that support modality specific language learning for spoken and signed language.

Functional magnetic resonance imaging (fMRI) data strongly supports the view that a core, modality independent, processing network, encompassing left dominant inferior frontal (IFG), superior temporal (STG) and inferior parietal (IPL) supports both spoken and signed language communication^4–9^ review^10^. The view that core language processing draws on innate and modality independent networks is further supported by the finding that similar patterns are observed in signers—both deaf and hearing—who acquired sign language in early childhood^6–8,11–13^ or as hearing late learners^14,15^.

The precise nature of these core language networks^16,17^ and, particularly, their delineation from modules that focus on more general auditory or visual signal reception or motor actions, however, is poorly understood^18,19^. Most brain areas that would be considered the ‘core language’ network, like the IFG and STG/STS, also make essential contributions to a wide range other cognitive tasks (IPL, review^10^; STG/STS review:^20^). Other areas, that are necessarily active during sign language processing, for example primary sensory cortices or specialised (face and motion) processing areas, do not appear perform functions that are specific to sign language (review^10^), although in the deaf population, the auditory cortex is active in the processing of sign language (review:^21^).

One way to operationally define whether brain areas make task-specific contributions to cognition is to test whether they adapt during learning. For L2 sign language, for example, one might not expect early visual areas, motion or face processing areas to specifically adapt for this new task^10^ if sign-language interpretation does not add additional processing demands on these areas. Core language areas, similarly, might not be expected to change if sign-language interpretation or execution shares common representations. Areas, that adapt to extract specific (visual) information, for example by representing minimal contrasts between gestures, however, would be expected to show changed functional activity as well as structure.

Learning-induced functional activity changes in the brain have been documented for a wide range of different learning tasks, such as music^22^, sport^23^, visual^24^ or language learning^25,26^ or the ventral visual cortex specifically, which comprises numerous regions involved in recognition of objects and non-object information and changes in this region can also be observed in response to non-visual sensory inputs^27,28^. The visual word form area, a part of the ventral region, as well as the dorsolateral prefrontal cortex, the primary motor cortex and the pre-supplementary motor area are involved in the processing of visual information regarding motor tasks. This makes it likely for them to be involved in sign-language learning^27,29^. These areas typically show increased BOLD response relative to rest after learning although same authors report reductions in activity, that may be linked to task difficulty and attentional factors^30,31^.

Spoken language learning also tends to lead to greater activity after training but evidence for functional activation changes scarce for sign language^32–34^. There, however, is no monotonic relationship between language learning and functional activation: Activation differences between first (L1) and second language (L2) depend on the relative proficiency for L2, with the largest difference seen for advanced learners, but little difference for speakers that are equally proficient in both languages^35^. Williams et al. (2016), showed the sequential involvement of putamen and supramarginal gyrus in an initial (‘phonological’) followed by IFG (‘lexico semantic’) later during L2 sign language learning in hearing participants over a year. Thus, sign language learning is likely to lead to fMRI increases in key areas, but these changes are not coincident across all brain areas involved and depend on proficiency.

Language processing draws on an extended network of functionally specific brain areas. In addition to functional changes, learning languages and bilingualism have been associated with increases in functional connectivity between these areas, notably between IFC and STG bilaterally^35^. These changes depend on initial language exposure^36^ as well as age^37^.

In additional to functional activation and connectivity changes, microstructural differences in white matter between deaf and hearing participants were found in a number of studies using Diffusion Tensor Imaging (DTI), a sensitive technique for measuring brain structure by quantifying the degree and directionality of water diffusion. DTI analysis produces four common measures: the mean diffusivity (MD) and fractional anisotropy (FA) as a measure of directionality, as well as the diffusivity along the main axis (axial diffusivity AD), and orthogonal to it (radial diffusivity RD)^38^. FA and MD are important measures of white matter integrity^39^. Higher FA and/or lower MD value are expected with successful learning or as a precondition of high performance^40,41^. Significantly lower FA and increased diffusivity (MD, RD, AD) are typically found in hearing related brain areas when comparing deaf and hearing individuals^42–44^, and these differences are reflected in behavioural performance^45^.

Brain microstructure, as measured by DTI, is an effective indicator for skills that range from musical aptitude^46^, performance^47^, reading skills^48^, as well as an indicator for neurological or neurodegenerative disease^49,50^.

Most language-related plasticity studies compare extensively trained groups controls or consider relatively long training durations. These studies commonly show that extensive training is linked to increased FA and decreased diffusivity measures (e.g.^26^; (language learning)^51^; (n-back memory), and^52^; (cognitive training), although some studies report opposite results (e.g.^47,53,54^.

Recent studies in our and other labs have shown diffusivity reduction and/or FA increases in task-relevant brain areas after very short training periods, Hofstetter et al.^55^), for a lexical task, showed rapid diffusivity reduction in language-related brain areas, while Tavor et al.^56^ showed that just 45 min of keyboard training causes significant MD reduction in motor areas. Similarly, training on memory tasks causes rapid changes in the hippocampus^57,58^. Tavor et al.^59^ showed that initial changes returned to baseline after approximately a week, suggesting that the observed effects may *not* be the result of direct changes to neural processes, but perhaps instead reflect changes that are precursors to more long-term neural changes.

In brief, short-term learning causes consistent increases in FA and/or decreases diffusivity metrics, even after very short-term training on new skills. These alterations frequently are correlated with performance and do not seem to be constrained by the age of the individuals involved^57,60–62^. We therefore expect increased FA and reduced MD, RD and AD measures as a result of L2 sign-language learning in areas that rapidly adapt.

### Hypotheses

Our hypothesis revolves around the notion that the acquisition of sign language skills in the short term, particularly among participants who have no prior familiarity with sign language, can lead to significant and discernible changes across multiple dimensions. These dimensions encompass behavioural, functional, and microstructural aspects, collectively offering a comprehensive understanding of the transformative effects of brief sign language training.

In our study, we anticipate that as short as 3 h training session focusing on the learning of new manual gestures will be sufficient to induce discernible alterations in both behavioural performance and neural patterns. This assumption is grounded in the belief that the human brain possesses remarkable plasticity, allowing for rapid adaptation and reorganisation in response to novel linguistic and motor challenges.

Specifically, our research aims to unveil a series of changes that extend across multiple domains. Firstly, we anticipate observing behavioural changes, manifested through participants’ improved sign language proficiency and communication skills. These changes may be evaluated through assessments of fluency, accuracy, and comprehension of sign language gestures.

Secondly, we hypothesize that there will be detectable functional changes within the brain, in particular, related to haemodynamic changes that can be assessed by measuring blood flow and oxygenation patterns during task performing.

Thirdly, we anticipate that changes in functional connectivity, as assessed through neuroimaging techniques, will occur. These alterations are likely to be observed in neural networks associated with language processing and communication, reflecting the brain’s adaptation to the new linguistic input.

Additionally, our investigation delves into the microstructural level of the brain, aiming to identify subtle alterations in white matter tracts. We anticipate that microstructural changes, such as increased FA and reduced diffusivity measures, will be evident in regions linked to language-related and visual-related processing. These alterations are anticipated to underscore the neurobiological underpinnings of the participants’ improved sign language skills..

## Results

### Behavioural performance

**Fig. 1:**
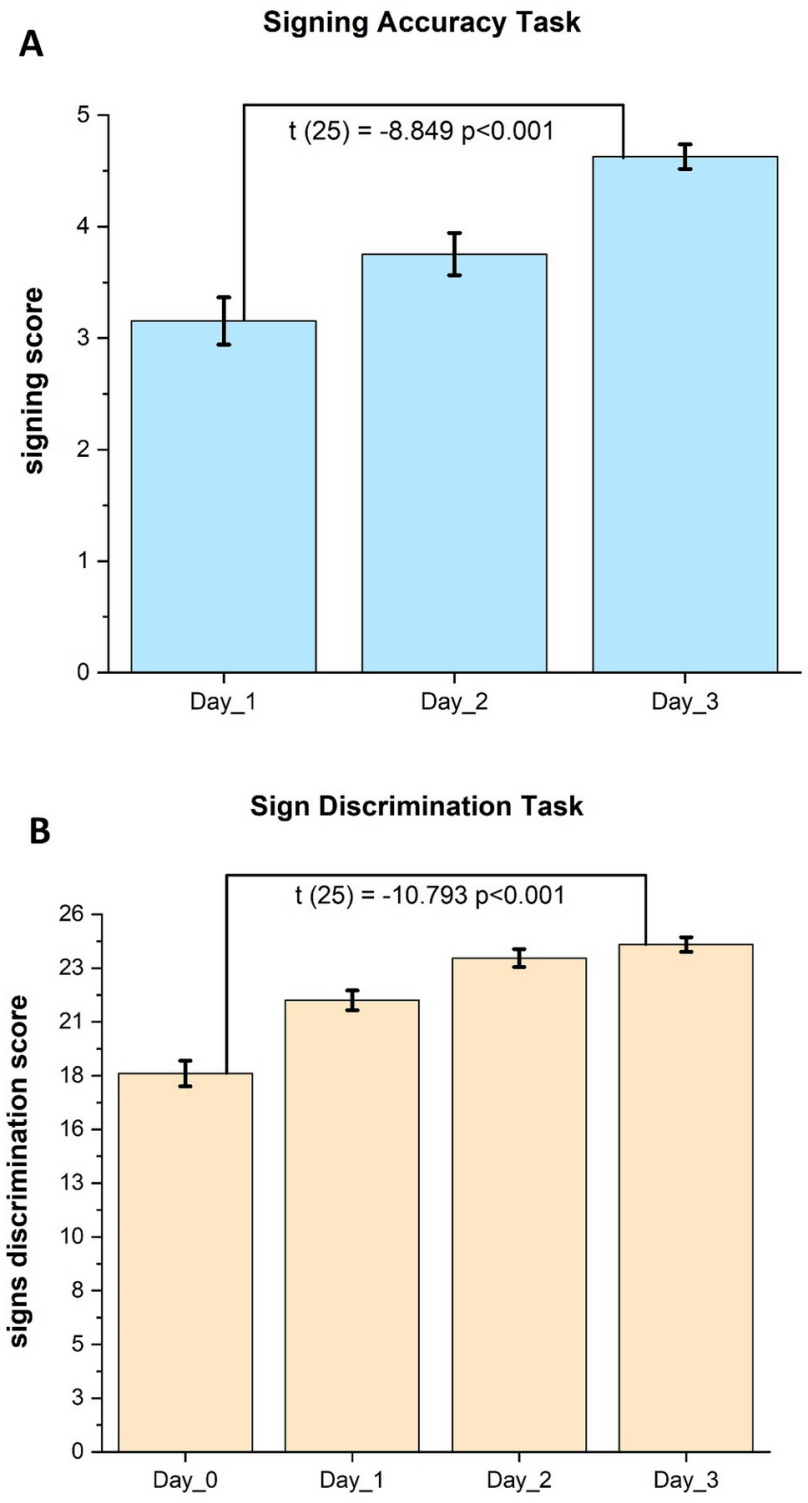
(**A**): Signing accuracy score (out of 5) shows significant improvement between day 1 and day 3 of training. (**B**): Signing discrimination score (out of 26) shows significant improvement between day 0 (before training) and day 3 of training.

Daily behavioural performance data were collected for the two valuables: signing accuracy and sign discrimination. The participants showed significant improvement in both tasks after training. The signing accuracy score significantly improved t(25) =  − 8.849, p < 0.001, from (m = 3.15/5.00, SD = 1.07) on day_1 to (m = 4.62/5.00, SD = 0.565) on day_3, as shown in Fig. 1A.

For the second task in Fig. 1B, significant improvement t(25) =  − 10.793, *p* < 0.001 was seen from day_0 (m = 18.30/26.00, SD = 3.14) to day_3 (m = 24.53/26.00, SD = 1.81).

### Functional results

#### Training-related brain activation changes

**Fig. 2:**
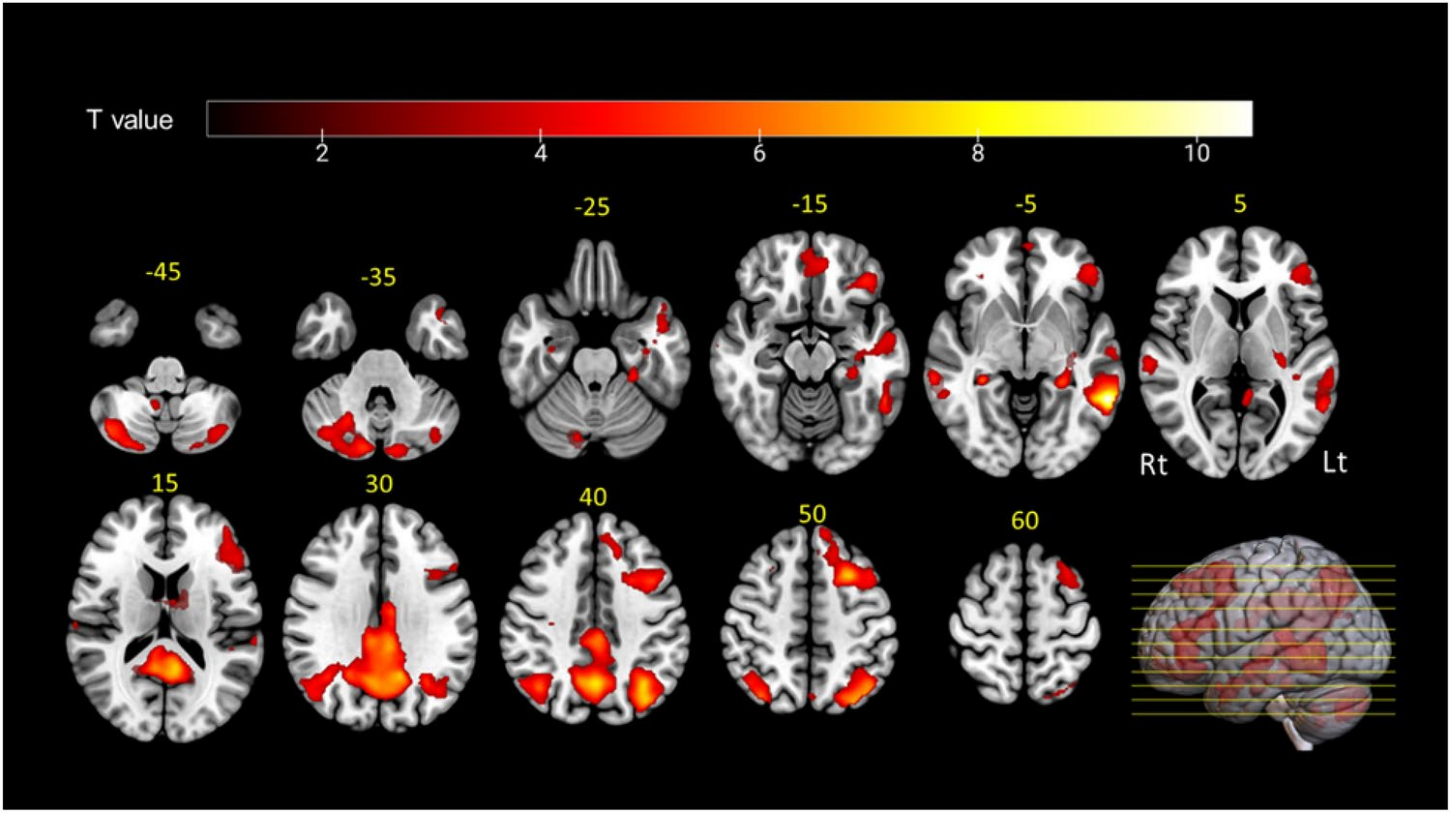
Training-related whole-brain analysis. Significant (p_(FWE)_ < 0.05 cluster level) fMRI signal increases after BSL training. Yellow numbers: axial plane slice number (z), Rt: right, Lt: left.

Whole-brain fMRI analysis was performed to determine the training effect (post > pre). Significant (p_(FWE)_ < 0.05 cluster level) BOLD signal increasing was seen in multiple language brain areas including bilateral middle temporal gyrus, left angular gyrus, left middle and inferior frontal gyrus (IFG), and right cerebellum (Fig. 2). Full details for all changing-activity areas are shown in Table 1.

**Table 1:** Results of fMRI whole-brain analysis after training. Regions, p value, cluster size, T value and peak MNI coordinates for all significant clusters. Abbreviations: Lt, left; Rt, right; MTG, middle temporal gyrus; STG, superior temporal gyrus; AG, angular gyrus; IPL, inferior parietal lobule; SPL, superior parietal lobule; PCgG, posterior cingulate gyrus; PCu, precuneus; MFG, middle frontal gyrus; IFG, inferior frontal gyrus.

No	Area	*P*_*(FWE)*_ cluster	Cluster size	MNI coordinate			
				T	x	y	z
1	Lt MTG	< 0.001	1260	10.63	− 60	− 50	− 6
	Lt STG			8.94	− 64	− 30	12
2	Lt AG	< 0.001	1762	8.19	− 34	− 62	38
	Lt IPL			7.89	− 30	− 72	47
	Lt SPL			6.9	− 22	− 70	48
3	Lt PCgG	< 0.0001	6907	8.09	− 10	− 50	18
	Lt PCu			7.95	0	− 62	40
4	Lt MFG	< 0.001	3064	7.58	− 26	14	50
	Lt IFG			7.55	− 44	42	13
5	Rt Cerebellum	< 0.001	1285	6.17	36	− 74	− 50
6	Lt Hippocampus	< 0.001	1158	6.1	− 26	− 38	− 4
7	Rt MTG	< 0.041	224	5.52	66	− 38	− 8
	Rt STG			4.65	62	− 29	9
8	Lt Cerebellum	< 0.002	460	4.85	− 12	− 84	− 38

#### Correlation between fMRI and behavioural performance

**Fig. 3:**
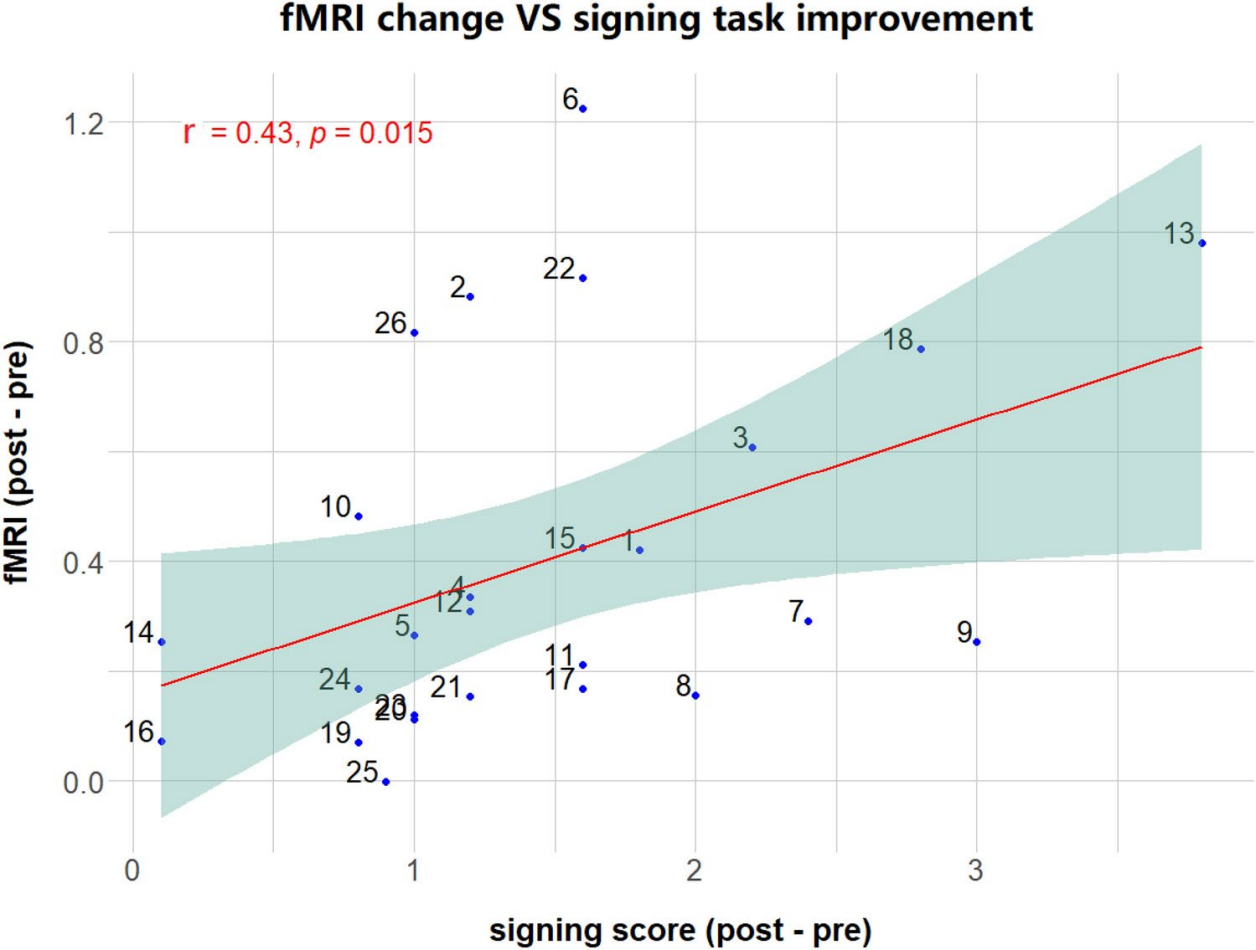
fMRI activation versus signing task improvement. The graph shows a significant positive correlation between the BOLD signal increasing in the right cerebellum and the participant’s progress in the signing task after training.

Behavioural improvement and functional brain changes were seen after training, we therefore expect the degree of these brain changes are correlated with learning improvement. fMRI signals were extracted from the brain areas that show neural changes. Positive significant correlation r(24) = 0.43, *p* < 0.015 was observed between the functional changes in the right cerebellum [x = 36, y = − 74, z = − 50] and performance improvement in the signing task as shown in Fig. 3.

### Functional connectivity

**Fig. 4:**
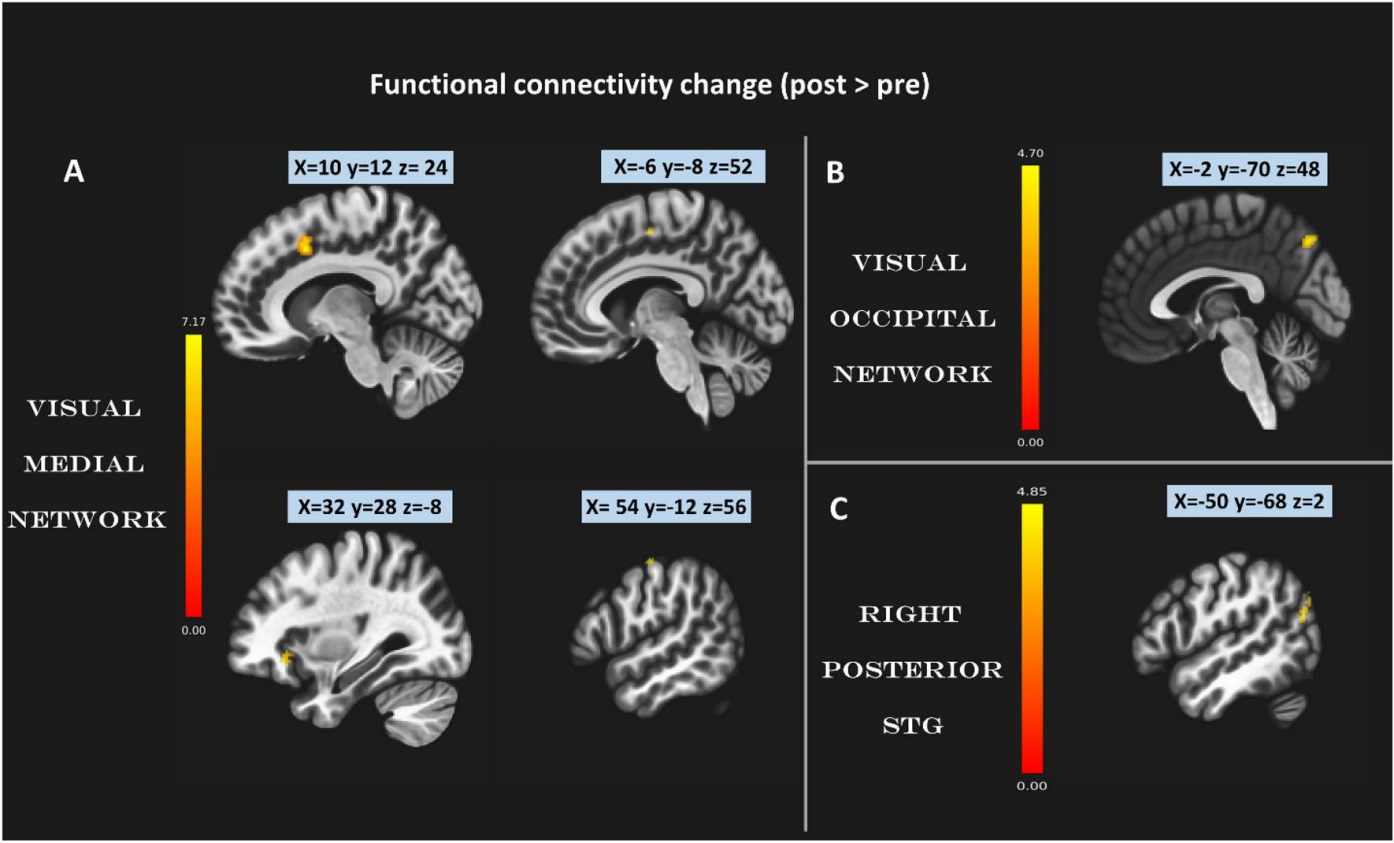
Training-related functional connectivity changes Three seeds show significant (p_(FDR)_ < 0.05 cluster level) increase in functional connectivity after training. Seed 1: Visual medial network seed in (**A**), Seed 2: Visual occipital network seed (**B**) and Seed 3: Right posterior superior temporal gyrus seed in (**C**). All coordinates given are in MNI standard space.

Seed-to-voxel analysis was performed to investigate the functional connectivity changes after training. Amongst all selected seeds, only three main seeds illustrated significant increase in functional connectivity after training with other voxels in the brain, as shown in Fig. 4:

1. The visual medial network seed with three brain areas; Rt frontal eye fields BA 8 [x = 10, y = 12, z = 24], Lt ventral anterior cingulate [x =  − 6, y =  − 8, z = 52], Rt IFG opercular part [x = 32, y = 28, z =  − 8] and Rt precentral gyrus [x = 54, y =  − 12, z = 56].
2. Visual occipital network seed, with Lt precuneus [x =  − 2, y =  − 70, z = 48].
3. Rt posterior superior temporal gyrus seed with Lt angular gyrus [x =  − 50, y =  − 68, z = 24].

Table 2 lists more details of seed coordinates and cluster sizes.

**Table 2:** Functional connectivity changes after training. Seed name, MNI coordinate, p value and cluster size for all significant clusters.

No	Seeds	Area	MNI (x,y,z)	Size (vx)	p-FDR
1	**Visual medial** [x = 2, y = − 79, z = 12]	Rt Frontal eye fields [BA 8]	10 + 12 + 42	311	0.0001
		Lt ventral anterior cingulate	− 6 − 8 + 52	106	0.000695
		Rt Inferior Frontal Gyrus (IFG). Opercular part	32 + 28 − 8	53	0.028625
		Rt precentral gyrus	54 − 12 + 56	48	0.033627
2	**Visual occipital** [x = 0, y = − 93, z = − 4]	Lt precuneus	− 2 − 70 + 48	59	0.040816
3	**Right posterior superior temporal gyrus** [x = 59, y = − 42, z = 13]	Lt angular gyrus	− 50 − 68 + 24	84	0.005313

### DTI results

#### Effect of training

**Fig. 5:**
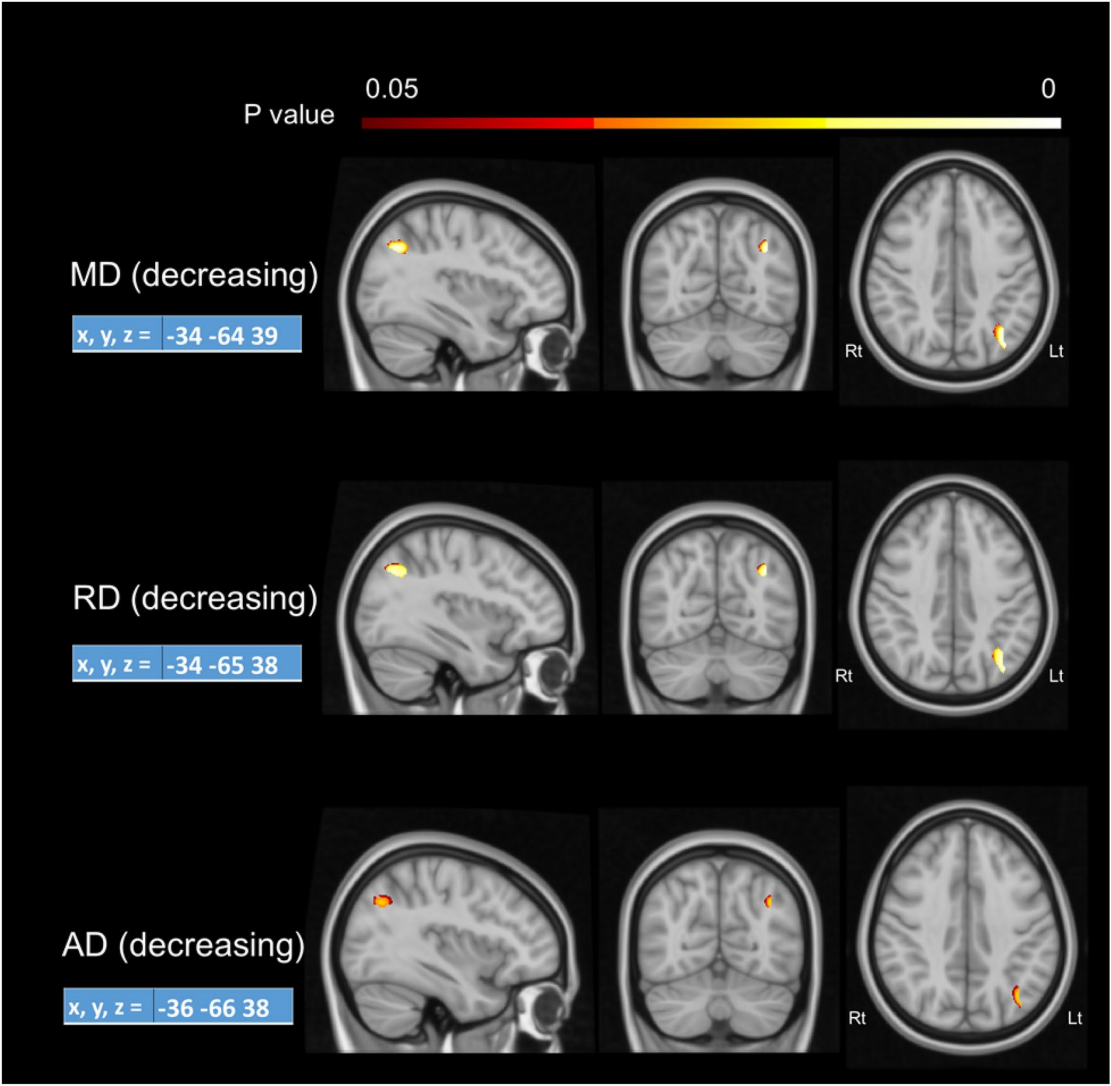
DTI changes after training. Reduction of MD, RD and AD in the left angular gyrus is significant. Rt: right, Lt: left.

**Fig. 6:**
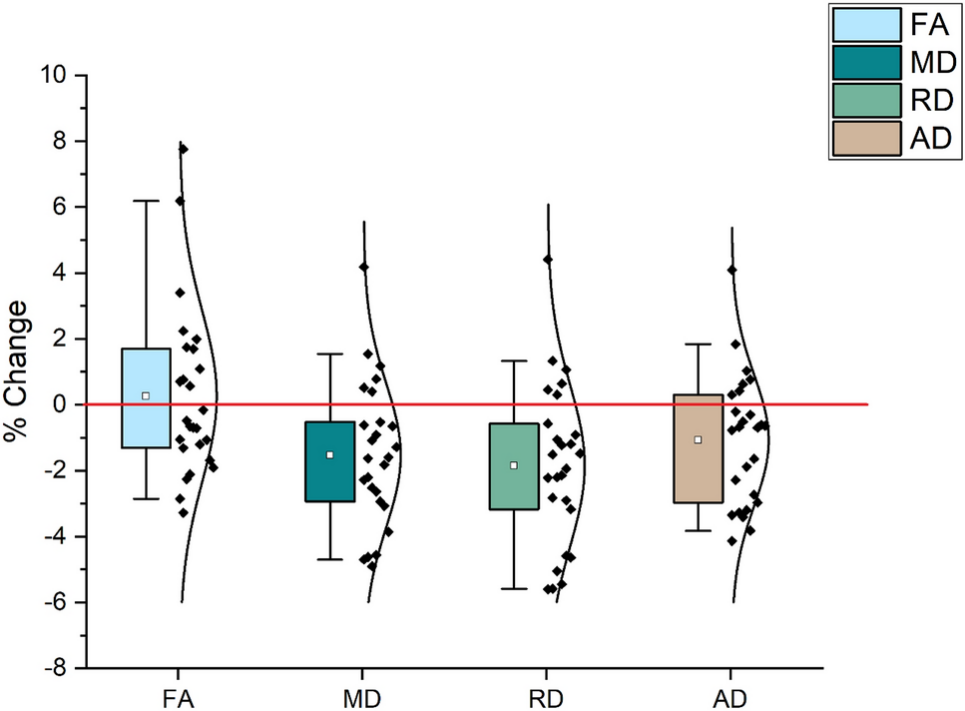
Data extraction of DTI values (training group). The graph shows the percentage change for all DTI measures, recorded in the angular gyrus, after training (increasing FA and decreasing MS, RD and AD). Black dots represent data from each participant, large coloured boxes are the 25–75th percentiles, of the data, small white boxes (mean), whisker are the 5 and 95 percentiles.

The principal prediction was that structural changes should be co-located with functional changes. Therefore, the fMRI training-related result was used as an ROI mask for DTI analysis. The prediction was confirmed for the training group by showing significant (*p* < 0.05) reduction (post-training − pretraining) in MD, RD and AD values in the Lt angular gyrus after training (Fig. 5). Data extractions from these clusters showed significant reduction after training for MD, RD and AD, respectively, t(25) = 3.626 *p* < 0.001, t(25) = 3.945 *p* < 0.001 control group’s results shown in Fig. 6. No significant FA changes t(25) =  − 0.492 *p* < 0.627 were observed in the target area after tinning for this group.

The results of the control group showed no significant changes for any DTI measures in the target area. For FA t(25) =  − 0026 *p* < 0.979, MD t(25) = 0.223 *p* < 0.0.825, RD t(25) = 0.011 *p* < 0.991, and AD t(25) =  − 0.460 *p* < 0.649. Appendix 3 shows the percentage changes for all of the DTI measures.

#### Correlation between DTI and behavioural performance

**Fig. 7:**
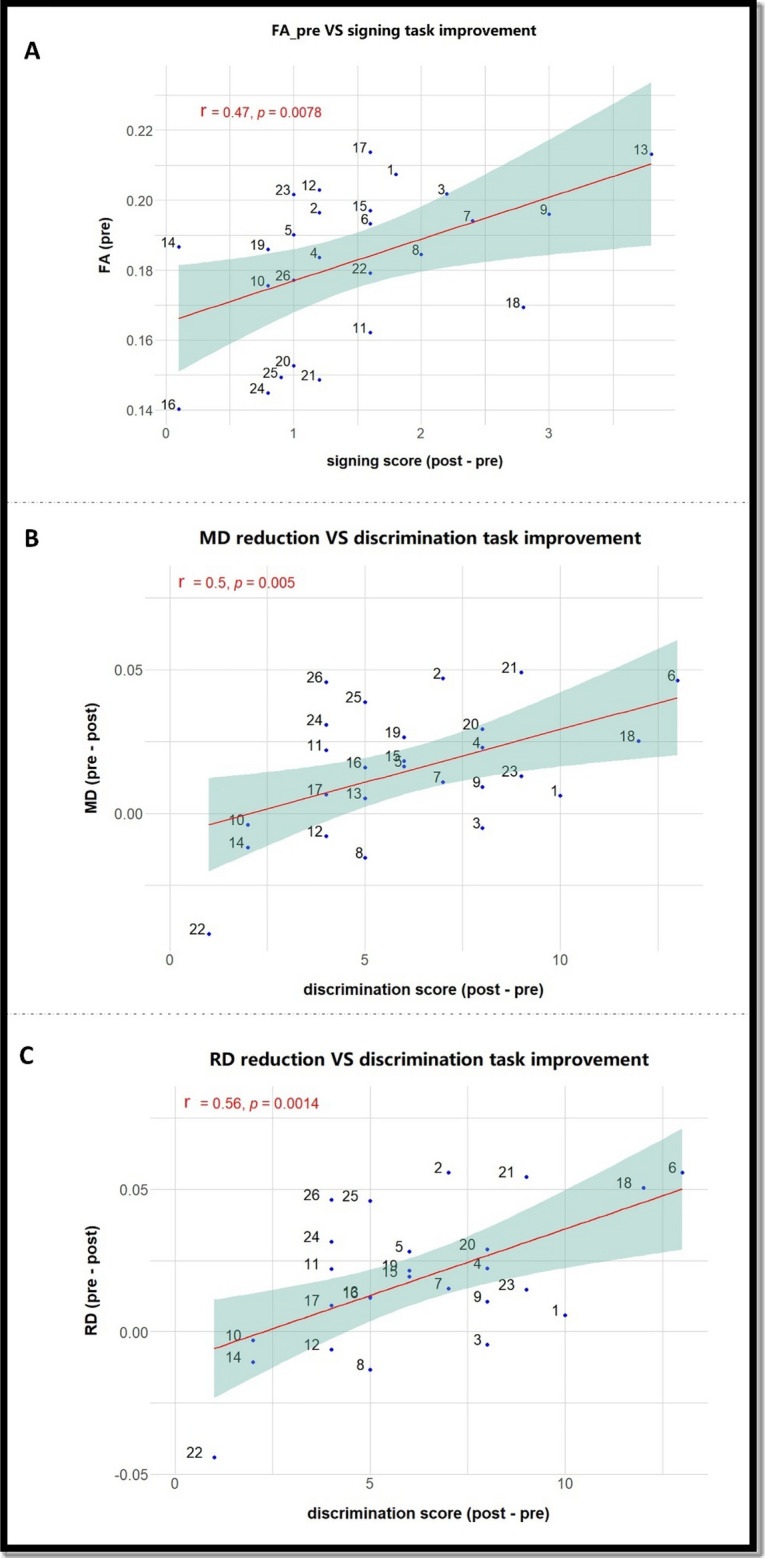
Correlation between DTI and behavioural data. (**A**): Pretraining FA values versus signing task improvement. The graph shows a significant positive correlation between FA value before training and the participant’s progress in the signing task. (**B**): MD change versus performance improvement in the discrimination task. A significant positive correlation is noted between changes in MD and the participants’ improvement in the discrimination task. (**C**): RD change versus performance improvement in the discrimination task. A significant positive correlation is noted between changes in RD values, and the participants’ improvement in the discrimination task.

Since microstructural brain changes were observed after training, we test whether the degree of these changes is correlated with learning improvement. DTI values were extracted from the brain regions that exhibited neural changes. Participants with high pretraining FA values performed better in the signing task. Figure 7A shows FA pretraining values were positively correlated with the participants’ behavioural improvement in this task r(24) = 0.47, *p* < 0.008. More so, positive correlation was also seen between *reduction* of MD and RD values (pre -post training) and behavioural *improvement* in the discrimination task. Figure 7B, C show significant value for MD r(24) = 0.50, *p* < 0.006) and RD r(24) = 0.56, *p* < 0.001 respectively.

## Discussion

In summary, this study aimed to uncover neural changes in response to the short-term learning of sign language in a group of healthy volunteers using fMRI and DTI. The results showed that sign language teaching was effective based on significant production and discrimination performance improvements in British sign language over the training period. This provided assurance that changes in neural activity detected with MRI are related to the learning process.

Data derived from fMRI revealed significant increases in BOLD signalling within key language regions including the left inferior frontal gyrus (Broca’s area), the middle temporal gyrus, the left angular gyrus, the left hippocampus, and the right cerebellum. These findings highlight the extensive interaction between key brain areas involved in sign language processing and indeed, enhanced BOLD activity in these regions have been previously reported. In one example^63^, after three months of sign language learning, showed significant BOLD response increases within left dominant inferior frontal gyri, the middle temporal gyrus, and other language areas. The present study consistently showed that BOLD signalling was enhanced largely in left-hemispheric language areas.

Van Ettinger-Veenstra et al.^64^ showed intense activation of the left angular gyrus, with extension to the temporal lobe and inferior frontal gyrus. While this study was based on sentence reading and not sign linguists, the findings may only have some applicability to the results of the present study, but they do support the involvement of the left angular gyrus in language processing and likely implicate this region in the language learning pathways. Previous studies have also shown that there is an activation of the left angular gyrus in response to non-signing language tasks, although activation was more intense for non-congruent and ambiguous tasks, as compared to congruent and unambiguous tasks^65–67^. To the authors’ best knowledge, no previous studies have investigated the role of the left angular gyrus in sign linguists or sign language learners using fMRI. Thus, the findings of this study contribute to the knowledge gap and may help to advance knowledge in the various pathways through stimulating and informing ongoing research in the field.

In line with this evidence, learning sign language is consistent with classically accepted activity within the inferior frontal cortex, but also highlights the role of other key areas involved in motor learning and memory including the inferior parietal lobule, superior parietal lobule, lateral occipital gyrus, and other temporo-occipital regions, which involve pathways regulating motion-related perception^5,15^. Plasticity within the parietal region has also been implicated in the working memory of sign language and the phonological interpretation of motor signs^9,68^. However, evidence implicating the parietal region in sign language learning has been mixed with Williams et al.^69^ finding no meaningful variances in activity at ten months post-learning using fMRI. Notably, in our study, these areas showed strong activation in all participants after a much shorter exposure to the material. Additionally, a key finding in^15^ study was the observation of specialisation for sign language processing with activation in the inferior parietal lobule (i.e., angular gyrus) for all learners, which aligns with our own observations.

Aloufi et al.^24^ demonstrated non-monotonic changes in MRI activity during the learning process. Initial increases in activity reduced over time, aligning with the idea that as participants become more proficient, the mental effort required for cognitive tasks decreases. This pattern is consistent with findings in functional imaging tasks and cognitive skill acquisition. Our study revealed increased neural activity, indicated by BOLD increasing, within the hippocampus, emphasizing its crucial role in spatiotemporal learning and memory processes. These functions are vital for sign language acquisition^70^, supporting previous research highlighting the hippocampus’s significance in language-related memory. Notably, we observed an increase in activity in the right cerebellum, and this was correlated with improvements in signing performance. This finding is in line with the established concept of cerebellar activation being contralateral to the dominant language side of the cerebral cortex^26,71,72^, both for spoken and sign language. In the context of spoken language, previous studies have documented the involvement of the right cerebellum in language-related tasks^73,74^. Similarly, our results align with prior research demonstrating increased right cerebellar activity in relation to sign language learning, particularly in response to verb signing^75,76^.

This study also showed that functional connectivity increased between the right posterior superior temporal gyrus and the left angular gyrus. This finding is consistent with previous research that highlights the role of the left angular gyrus in language processing and integration within the semantic system and the default network^77^. The increased connectivity observed in our study suggests that the left angular gyrus plays a crucial role in the integration of multisensory information, which is essential for the comprehension and production of sign language. This aligns with findings by Vasileiadi et al.^78^, who demonstrated the importance of functional connectivity between language-related brain regions in processing complex linguistic tasks. Our results contribute to the understanding of the neural mechanisms underlying sign language learning and emphasize the significant role of the left angular gyrus in this process.

Although the left angular gyrus was not explicitly localised in the parietal lobe in the study by Berken et al.^36^, this region is critical to language learning and function given its role in semantic processing^79^. Sign language processing has also been recently reviewed in a meta-analysis^80^ highlighting that task performance is a strongly linked to activity across a left dominant network encompassing frontal, temporal and occipital regions, and that similar activity is observed in spoken and written language processing. Several authors have posited that the left supramarginal gyrus of the parietal lobe is necessary for phonological interpretation in sign language and that the frontal, middle and superior temporal cortices are needed for syntactical processing^81^. The design of his study does not allow specific regions to be linked to syntactical versus non-syntactical processing in sign linguists, but this could form a key component of follow-on research. Wider previous research has also investigated the influence of the age of language acquisition upon neural processing, which could also be determined in any follow-on research into the short-term neural and plasticity effects in sign language learners.

Our training protocol was designed to provide participants with an introductory understanding of BSL through a combination of discrimination and signing tasks. The discrimination task focused on the participants’ ability to distinguish between similar signs, while the signing task assessed their ability to accurately reproduce BSL sentences. Importantly, the instructions on the cards for the signing task were designed to engage participants in meaningful sentence production. Each card presented a written sentence that participants needed to comprehend and then sign, thus incorporating a semantic component into the task. This approach ensured that linguistic processing was involved in the learning process. Furthermore, while our study may not cover the full complexity of comprehensive sign language learning, it aimed to investigate the neural adaptations associated with the early stages of BSL acquisition. Both discrimination and signing aspects of learning were included to provide a general view of the initial learning phase. The significant improvements in both tasks observed in our study suggest that participants engaged in meaningful language processing, rather than pure motor sequence learning.

Imaging using DTI showed that there were significant decreases in all diffusivity measures (MD, RD and AD) within the left angular gyrus post-learning. Notably, behavioural improvements were significantly correlated with DTI measures, mostly MD and RD. This provides strong evidence that the observed changes are indeed learning-related.

A significant positive correlation between FA baseline values and performance improvement in the signing task is a valuable measure. It suggests that the initial FA values can provide predictions for future learning outcomes and accords with findings that FA can predict reading skills^48^, gymnastics^82^ and musical performance^47^.

Based on the tasks that were used in this study, increasing activity in the left and right superior temporal gyri is an expected result. The role of these areas during action observation and language categorization is confirmed in earlier studies^83–85^. Additionally, implicating the middle temporal gyrus, and likely the visual word form area, in sign language learning, is a plausible observation in view of the temporal cortex being recruited to support the processing of visual as opposed to auditory information^86^. While lateralisation of neural activity in language has been related to hand dominance in some studies, the evidence is somewhat mixed with laterality having been found to occur independent of hand dominance^87^. Thus, it is unlikely that hand dominance variance in this study would have confounded or mediated the effects reported and thereby, cerebellar activation may occur independently of the motor component of signing. Clearly however, the visual cortex plays a role in the reception of information being taught to learners of sign language and although activity was not determined in this region in this study, prior research has shown that the V1, V2 and V5 domains of the visual cortex observe heightened activation in signers^88,89^. Despite this, the lateralisation of activation of key language areas, including the visual cortex, encounters variance based on age at sign language development, which affirms the influence of age of sign teaching exposure^87,89^.

Overall, this study has provided valuable insights into the dynamic nature of neuroplasticity during the process of learning sign language within a short-term training period. Our findings add to the growing body of literature that enriches our understanding of the neuroplastic mechanisms supporting sign language acquisition. By shedding light on the interplay of cognitive processes and neural responses, our research advances the field’s knowledge of how the brain adapts and learns to process sign language in a relatively brief training period. Longitudinal fMRI studies, such as ours, are particularly powerful in detecting within-subject changes in brain activity associated with specific training or learning interventions. By comparing pre- and post-training scans within the same individuals, we control for individual variability, focusing on neural changes directly related to the intervention, which in this case was the learning of British Sign Language (BSL). The significant improvements observed in both sign discrimination and production accuracy, along with corresponding neural changes, strongly suggest that these adaptations are related to BSL learning.

While our within-subject design enhances the sensitivity to detect these training-related effects, including a control group trained on non-linguistic gestures could further clarify the extent to which observed changes are specifically due to linguistic learning versus general motor learning. The classification and replication of manual motor actions linked to linguistic constructs, as performed in our behavioral tasks, could theoretically occur outside of explicit language processing. While such mapping is essential in language, much like phonetic categorization in spoken language, these processes may not be language-exclusive. Due to the high cost of MRI scans, we could not include this additional control group in the current study; however, we recommend this in future studies to more precisely delineate the specificity of neural adaptations to linguistic versus non-linguistic training. It is important that future research continue to explore the varied neural activation pathways associated with learning sign language in both early and late signers, in order to elicit the time-by-exposure-related variances in the processing of sign language information.

## Methods

### Participants

Twenty-six (11 males and 15 females) healthy native English-speaking participants were included in the study; their mean age was 22.76 years, SD = 3.50, range (19–36). All self-reported to be right-handed and having no previous exposure to sign language. They also reported normal hearing and normal or corrected to normal vision. As part of the screening procedure for the MRI scans, participants declared not to have any history of neurological or psychiatric disorder. No incidental findings were discovered during the routine neurological assessment of the MRI images. Ethical approval was obtained from the research ethics committee of the University of Liverpool (application number: 3384). We confirm that all experiments were performed in accordance with relevant guidelines and regulations. Prior to participation, written informed consent was obtained from all participants, and all received payment to take part in this study.

A matching control group of 26 (12 males and 14 females) healthy participants was used as a control group for the structural analysis. Mean age = 25.91 years, and SD = 4.10, range (19–35). This control group is a part of a separate study focusing on non-linguistic training, the results of this group will be published separately.

Two MRI scans were taken using the same imaging parameters in the same scanner. This group trained to perform an auditory task, that does not require language: audio pitch discrimination. The training period for this group was four consecutive days, and the two MRI scans was taken just before and immediately after the training.

### Study design

A pretraining MRI scan was taken for all participants 1 day before the training began. Then, participants were interactively trained over three consecutive days (1 h per day). The post-training MRI scan was taken 1 day after the last training day (Appendix 1).

#### Materials

During the three training sessions, the participants learnt how to sign 26 sentences of minimal pairs of from BSL (Appendix 2). The signs in the two sentences in each pair are very similar in movement (left right) and location (torso), and they differ only in one sign. For example, one pair consists of sentence 1: me meet you tomorrow and sentence 2: me near you tomorrow. The subtle difference between *meet* and *near* is signalled by the position of the index fingers of both hands. In meet, index fingers are facing each other during the hand movement, whereas in near, they are not.

The training sessions were conducted online via Zoom platform by a qualified BSL deaf teacher. A qualified hearing interpreter attended all sessions to voice the signs and explain the procedure. The participants were spread into nine subgroups (two to three participants per group) to be accommodated in the MRI scanning centre appointments over nine weeks.

#### Training protocol and performance assessment

The teacher started each training session by going through the list (as pairs), and the participants were instructed to watch and spot any difference between each pair. Then, the teacher went through the list again but twice for each pair. For the first time, the participants just watched and were told the difference. For the second time, they were asked to copy the signs. On the two following training sessions, they were asked to sign the full list and perform extra practice exercise.

Improvement assessment was measured immediately after each training session and was quantified by two behavioural variables. Variable (1) is performance on a sign discrimination task, where the participants were asked to identify the difference between the signs in each of the 26 pairs. Short video clips were recorded for the teacher whilst he signed the sentences. Half of clips contained two identical sentences with the same signs and meanings. The other half contained two minimal pair sentences (very similar signs that convey different semantics). For this variable, a computer-based forced-choice test was designed using PsychoPy V3.0.0^90^ software. The video clips were shown in a random order, and participants’ responses was recorded automatically. The participants task here was to tell if the two sentences have the same meaning or are different. Answers were given by pressing (S) for same or (D) for different on the computer keyboard.

For variable (2) the signing (production) accuracy for each participant was rated by the teacher. In this assessment, five different cards were visually (voicelessly) shown to each participant. Each card had one sentence to sign on. Thus, each participant had to understand and then sign the written sentences. The teacher watched and scored each signed sentence from 1 to 5 (1 = poor, 2 = fair, 3 = average, 4 = good and 5 = excellent). Then, the daily mean score for each participant was calculated.

### MRI acquisition

A Siemens Magnetom Prisma 3T MRI scanner equipped with 32-channel receiver head coil was used to collect images for this study. The MRI scanning protocol consisted of a T1-weighted (T1), fMRI and DTI.

To normalise the data spatially and complete tissue segmentation, anatomical 3D T1-weighted Magnetisation Prepared Rapid Acquisition Gradient Echo (MPRAGE) was taken for each participant. T1 imaging parameters included repetition time (TR) = 2000 ms, echo time (TE) = 2.26 ms, inversion time (TI) = 900 ms, field of view (FOV) = 256 mm, flip angle = 8° voxel size = 1.0 × 1.0 × 1.0 mm and slice thickness: 1mm.

In the fMRI run, the sign discrimination task (variable 1) was used as stimuli. The short video clips were placed randomly in a 780-s long block design. Each 15-s block task was followed by a 15-s rest. To acquire the functional scans, Echo-Planar Imaging (EPI) sequence with an advanced Simultaneous Multi-Slice (SMS) feature was used with the following settings parameters: TR = 1500 ms, TE = 30 ms, flip angle = 90°, FOV = 192 × 192 mm, slice thickness = 2.7 mm and interslice gap = 10%.

In order to correct the distortion in the DTI scan with no signal loss, two DTI sequences were acquired in opposite encoding directions^91^. DTI parameters were TR = 3200 ms, TE = 90 ms, FOV = 220 mm, diffusion directions = 64, slice number = 50, slice thickness = 2.5 mm and voxel size = 2.5 × 2.5 × 2.5 mm.

### Analysis

#### Statistical analysis

Considering the data were collected longitudinally over two time points, paired sample t-test were applied for the behavioural, functional and structural data for the training group and structural data from the control group. IBM SPSS 27.0.1 software was used to conduct the statistical analysis.

A Pearson correlation two-sided significance level of p = 0.05 test was used to measure the linear relationship between the behavioural performance in both tasks and the change in fMRI signal and DTI values.

#### fMRI analysis

fMRI data analysis was performed by using statistical parametric mapping software SPM12 (The Wellcome Department of Imaging Neuroscience, University College London, UK) running on MATLAB_R2020b (The Mathworks, Inc., Natick, MA, USA). The default preprocessing batch (preproc_fmri.m) was used in the preprocessing steps. For the slice-time correction, slice-timing vector and the reference time in milliseconds were used for all volumes. Then, all scans were spatially realigned to the first volume, normalised to the Montreal Neurological Institute (MNI) template, resampled into 2 × 2 × 2 mm cubic voxels and then smoothed using an 8 mm FWHM Gaussian kernel.

At the first level of analysis, a General Linear Model (GLM) approach was used to create the design matrices of the onsets and duration of both events: the baseline condition (rest) and the experimental condition (task). The images of each condition were combined together to create a single contrast file image. This process was carried out twice for each participant, pre- and post-training.

A second level of analysis was obtained to investigate the training effect for the whole group. All contrast images of the first level of analysis were combined together to create group level rest and group level task images. Paired sample t-test was employed to compare the contrast images for each condition at the two time points.

The REX toolbox for MATLAB (https://www.nitrc.org/projects/rex/) was used to extract the mean BOLD data from voxels with significant activations. For image visualisation, MRIcroGL software (https://www.mccauslandcenter.sc.edu/mricrogl) was used, and all coordinates were in MNI standard space. SPM12 anatomy toolbox (www.fz-juelich.de/inm/index^92^, was used to label the brain areas.

#### Functional connectivity analysis

The fMRI pre-processed data were fed in the functional connectivity CONN toolbox (www.nitrc.org/projects/conn, RRID:SCR_009550) to perform the functional connectivity analysis. In order to remove residual physiological effects, subject motion, and other potential confounders and outliers from the BOLD signal, the default denoising pipeline was applied^93^. Then, Generalised Psycho-Physiological Interactions (gPPI) maps were employed to modulate the training task at the first level of analysis. From CONN’s default seed ‘networks’, all areas related to language, vision and memory were identified as seeds at the first level of analysis. At the second level of analysis, the contrasts for each condition were set at the two time points, pre- and post-training, by using a GLM. Areas of significant values were defined by cluster threshold: *p* < 0.05 cluster-size p-FDR corrected, voxel threshold: *p* < 0.001 p-uncorrected^94^.

#### DTI analysis

All DTI data were pre-processed using the standard preprocessing pipeline of FMRIB Software Library (FSL 6.0.0, University of Oxford) according to Smith et al.^95,96^ as follows. Firstly, distortion was corrected for the two inverse scans by using the topup function. A binary individual brain mask was created for each scan, and then, eddy currents and subjects’ motion were corrected by the eddy_correct function. Lastly, fitting the diffusion tensors (λ1, λ2 and λ3) and FA were done by DTIFIT tool. Calculation of RD value was done by taking the average of λ2 and λ3. RD = (λ2 + λ3)/2^97^.

Following Schwarz and colleagues’ guide^98^, Advanced Normalization Tools (ANTS) software^99,100^ was used to perform voxel-based analysis for each DTI scan in the native space. Using ANTS, antsMultivariateTemplateConstruction2.sh command FA template images were generated for each subject. Then, in-native space realignment was done for all DTI maps (FA, MD, AD and RD) on the FA template. After that, the difference between pretraining and post-training scans was computed in native space by using the fslmaths command^24^. Each FA template was registered into a standard space (FMRIB58_FA_1mm.nii.gz) by antsRegistrationSyNQuick.sh function.

Using the same transformation, MD, RD and AD maps were transformed using antsApplyTransforms, and all images were then smoothed with a 5 mm Gaussian kernel^98,99^. The FSL-randomise tool (https://fsl.fMRIb.ox.ac.uk/fsl/fslwiki/Randomise^101^) with 5000 permutations was used for statistical inference. As the difference between pre- and post-training scans was computed initially, one sample t-test was performed.

To investigate the structural changes within the areas that showed functional changes, fMRI-guided mask was created for all clusters that survived the significant level p(_FWE_) < 0.05. Coalignment was performed for this mask to equate the FSL normalised spaces.

### Ethics approval

The study was approved by the central research Ethics committee at the University of Liverpool [ref number 3384]. We confirm that all experiments were performed in accordance with relevant guidelines and regulations.

### Consent to participate

All participants gave informed written consent and received payments to participate in the study.

## Supplementary Information

Supplementary Information.

## Data Availability

The datasets used and/or analysed during the current study are available from the corresponding author on reasonable request.
