# Supplementary material for: Neural adaptations in short-term learning of sign language revealed by fMRI and DTI
Source: Sci Rep. 2025 Feb 13;15:5345. doi: 10.1038/s41598-024-84468-z (PMC11825837; doi:10.1038/s41598-024-84468-z)
Supplement: Supplementary file 1 — Supplementary Information. [file 41598_2024_84468_MOESM1_ESM.docx]

**6 Supplementary Material**

Appendix 1


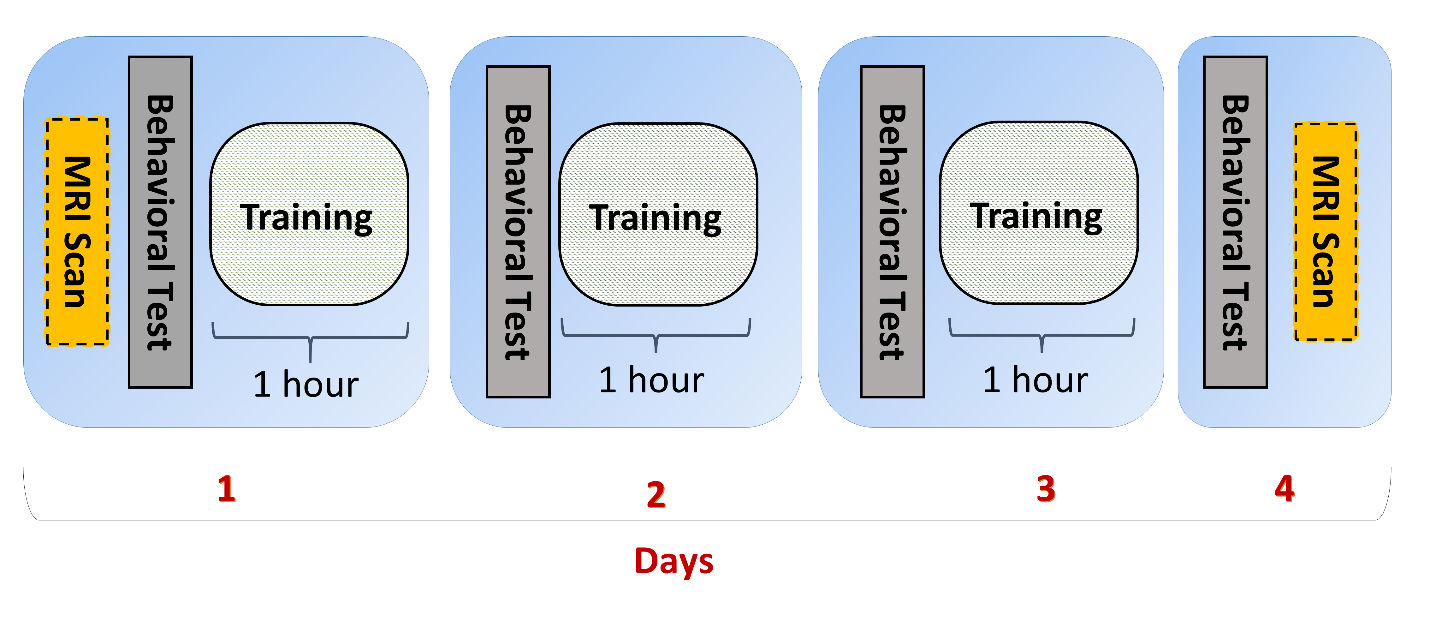


***Appendix 1.*** *The study timeline shows the protocol of the study including behavioral tests, training and MRI scans for all visits per day (red).*

Appendix 2

| 1. **Girl (RP) Star** | **Girl (RP) Bird** |
| --- | --- |
| 1. **Snow yellow** | **Snow possible** |
| 1. **Me finish exam** | **Me at last (boo) exam** |
| 1. **Me like mouse in garden** | **Me like try in garden** |
| 1. **He was angry** | **He was monkey** |
| 1. **Me went bed evening** | **Me went bed dark** |
| 1. **Me like colour red maybe** | **Me like colour red which** |
| 1. **Me went play with friends** | **Me went where with friends** |
| 1. **Me have bed upstairs** | **Me have bedroom upstairs** |
| 1. **Me can’t unfortunately** | **Me can’t do** |
| 1. **Me don’t like cruel** | **Me don’t like meat** |
| 1. **Me meet you tomorrow** | **Me near you tomorrow** |
| 1. **Me arrive Spain plane** | **Me arrive Spain maybe** |
| 1. **Me go shop food cheap** | **Me go shop food reduce** |
| 1. **Person who?** | **Person what?** |
| 1. **Arrive work easy** | **Arrive work hard** |
| 1. **Me want service** | **Me want delivery** |
| 1. **Boy arrive school bus 14** | **Boy arrive school bus 4 o’clock** |
| 1. **Daughter 18 years old today** | **Daughter out today** |
| 1. **Me don’t like trouble** | **Me don’t like cake** |
| 1. **Me like pictures (cinema) watch** | **Me like night watch** |
| 1. **I like castles** | **I like cauliflower** |
| 1. **Boy (RP) lovely today** | **Boy (RP) breakfast today** |
| 1. **Me meet friend yesterday** | **Me meet friend girl** |
| 1. **Me love red sweets** | **Me love chocolate sweets** |
| 1. **Me bought new coat** | **Me bought spring coat** |

***Appendix 2.*** *The learning task of 26 26 sentences from BSL. Minimal pairs in (red).*

Appendix 3


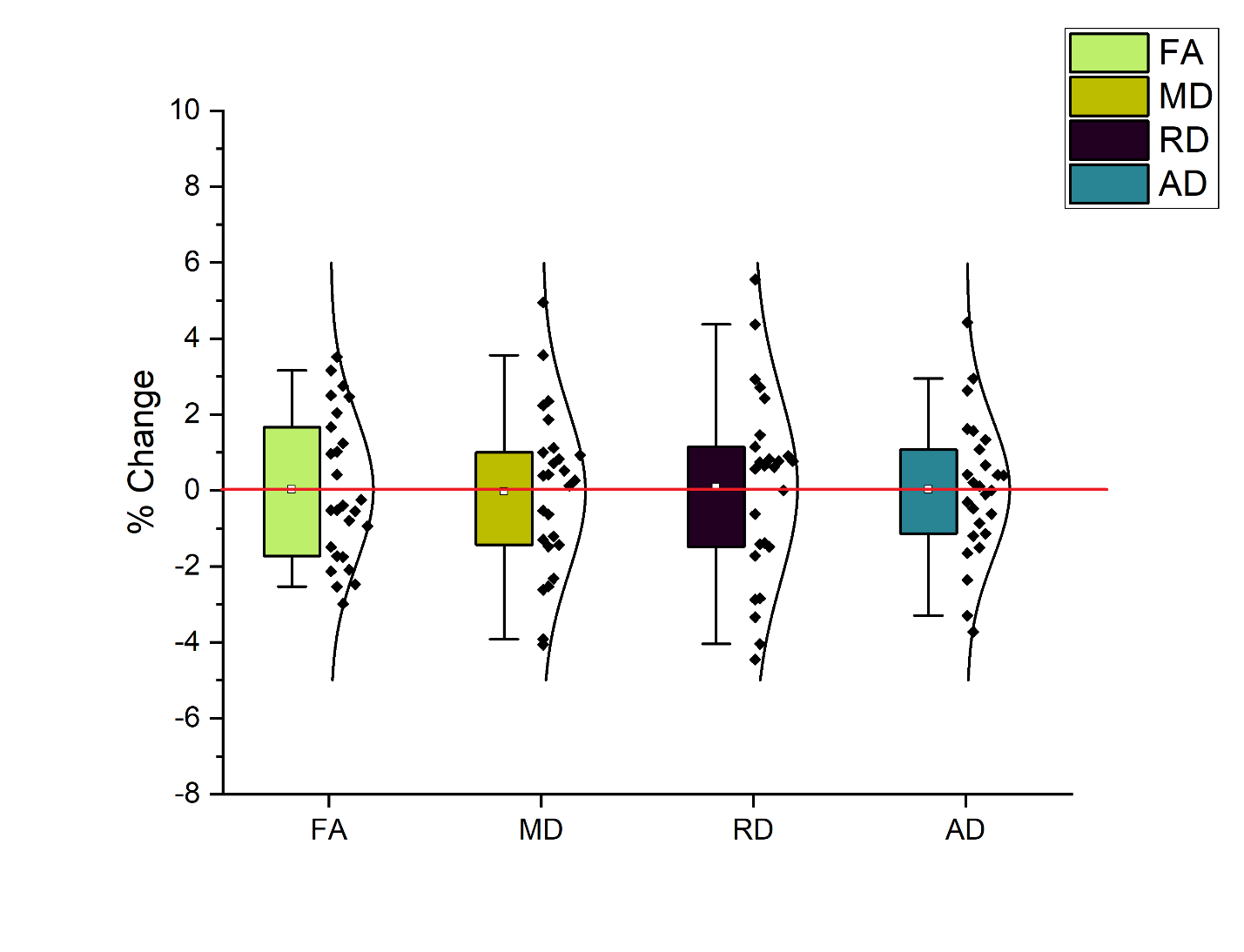


***Appendix 3****.* ***Data extraction of DTI values (control group)***

*The graph shows changing percentage of all DTI measures over time. Black dots represent data from each participant, large coloured boxes are the 25th-75th percentiles, of the data, small white boxes (mean), whisker are the 5 and 95 percentiles.*
